# Annealing Effect on the Properties of Electrochromic V_2_O_5_ Thin Films Grown by Spray Deposition Technique

**DOI:** 10.3390/nano10122397

**Published:** 2020-11-30

**Authors:** Kyriakos Mouratis, Ioan Valentin Tudose, Andrianna Bouranta, Cristina Pachiu, Cosmin Romanitan, Oana Tutunaru, Stelios Couris, Emmanouel Koudoumas, Mirela Suchea

**Affiliations:** 1 Center of Materials Technology and Photonics, School of Engineering, Hellenic Mediterranean University, 71410 Heraklion, Crete, Greece; kmuratis@hmu.gr (K.M.); tudose_valentin@yahoo.com (I.V.T.); mpandrianna@gmail.com (A.B.); 2 Department of Electrical and Computer Engineering, School of Engineering, Hellenic Mediterranean University, 71410 Heraklion, Crete, Greece; 3 Physics Department, University of Patras, 26504 Patras, Greece; couris@iceht.forth.gr; 4 Chemistry Department, University of Crete, 70013 Heraklion, Greece; 5 Institute of Electronic Structure and Laser, Foundation for Research & Technology-Hellas, 70013 Heraklion, Crete, Greece; 6 National Institute for Research and Development in Microtechnologies-IMT Bucharest, 126A, Erou Iancu Nicolae Street, 077190 Voluntari-Bucharest, Romania; cristina.pachiu@imt.ro (C.P.); cosmin.romanitan@imt.ro (C.R.); oana.tutunaru@imt.ro (O.T.)

**Keywords:** electrochromic V_2_O_5_, spray deposition, nanostructured metal oxides

## Abstract

Nanostructured electrochromic V_2_O_5_ thin films were prepared using spray pyrolysis technique growth at a temperature of 250 °C using air-carrier spray deposition, starting from ammonium metavanadate precursor in water, followed by annealing at 400 °C in O_2_ atmosphere for 2 h. The V_2_O_5_ films were characterized by X-ray diffraction, scanning electron microscopy, and Raman spectroscopy, and their electrochromic behavior was studied using optical spectroscopy and cyclic voltammetry in both the as-deposited and postannealing case. The studies showed that the simple, cost -effective, suitable for large area deposition method used can lead to an interesting surface structuring with large active surface properties suitable for electrochromic applications. Further studies for growth optimization and improvements of films properties and stability are to be performed.

## 1. Introduction

Vanadium pentoxide is the highest oxidation state 5+ of vanadium, and it has been known for more than a century. The structure and morphology of vanadium oxide films are intimately related to the deposition method and the operating conditions. As the direct growth of crystalline V_2_O_5_ films is very difficult except in the cases of some substoichiometric VO_x_ oxides, an annealing process of the as-prepared films is required in air and high temperature. The choice of a deposition method depends of the thin-film application. For example, electrodeposition [1], reactive sputtering [2], sol–gel [3], hydrothermal method [4], and doctor-blade route [5] are the most usually used methods for electrochromic V_2_O_5_ films that requests large surface coatings. During the deposition process in vacuum or in a reducing atmosphere, the removal of oxygen atoms occurs from the film network when V_2_O_5_ is heated above its melting point that induces the formation of defects or reduced VOx phases. Consequently, the structural and morphological disorders and phase instability can be controlled using appropriate deposition parameters. An excellent review regarding deposition techniques suitability for specific application and the history of V_2_O_5_ use was published by Beke [6] in 2011. Spray pyrolysis is considered as a cost-efficient technique and has been widely employed to fabricate V_2_O_5_ thin films. The usual fabrication of V_2_O_5_ films by spray pyrolysis involve over 400 °C substrate temperatures during deposition [7,8,9,10,11,12,13].

The present work concerns a follow-up of our recent preliminary results on a new approach for developing nanostructured V_2_O_5_ thin films for electrochromic applications reported in a Letter to Materials journal [14]. In particular, air-carrier spray deposition at quite low temperature has been employed with ammonium metavanadate dissolved in water. Mixed nanostructured granular and wall-like structures films, with homogeneous distribution over the film surface were obtained, and their properties were analyzed. The published study was limited to obtaining the specific thin-film structuring with suitable properties for electrochromic applications. The present study regards the effects of thermal treatment of the specific thin films and their properties evolution with respect to their electrochromic behavior in a successful attempt to use spray deposition at low temperature to define growth of the films 3D architecture followed by controlled annealing to achieve stoichiometry and consequently, electrochromic stable properties. It was observed that lower temperature growth, leads to a novel unreacted precursor-containing material structuring onto the substrates surface. This consists on walls-like structures and smaller granular covered valleys that can be improved by postdeposition annealing in O_2_ atmosphere. This kind of growth leads to the formation of a large active surface that favors ionic insertion and enhanced electrochromism. Up to our knowledge, no such approach for V_2_O_5_ fabrication was reported before.

## 2. Materials and Methods

A custom-made spray pyrolysis technique was used for the preparation of the samples, with which the deposition was carried out at a substrate temperature of 250 °C. The precursor solution was prepared by dissolving the required amount of ammonium metavanadate NH_4_VO_3_ (Sigma-Aldrich, St. Louis, MO, USA, ≥99.0% purity) in distilled water to have 0.01 M concentration. Four different quantities of solution 5, 10, 15, and 20 mL) were used to grow a series of V_2_O_5_ thin films with different thicknesses. The volume of solution defined the films thickness, which cannot be accurately measured. The deposition time is increasing as the volume of solution increases while the air carrier flow is constant. The nozzle-substrate distance was kept constant at 30 cm and gas pressure at 3 bars. After the deposition, the films were annealed for 2 h in an oxygen atmosphere at 400 °C.

X-ray diffraction (XRD) (Rigaku Ultra high-resolution triple axis multiple reflection SmartLab X-ray Diffraction System, Tokyo, Japan) was used to analyze the crystallinity of the deposited V_2_O_5_ films. The grazing incidence X-ray diffraction (GIXRD) patterns were recorded from 5° to 95° with a speed of 5°/min. Scanning electron microscopy (SEM) characterization was performed using a W filament LV6064 SEM (Jeol company, Tokyo, Japan) of the as-deposited films, and a field-emission scanning electron microscope (FE-SEM) Nova NanoSEM 630 (FEI Company, Hillsborough, OR, USA) for the anneald samples. All samples were characterized in high vacuum mode without any additional coating. Raman spectroscopy was performed at room temperature using a WITec Raman spectrometer (Alpha-SNOM 300 S, WITec GmbH, Ulm, Germany) using 532 nm excitation, from a diode-pumped solid-state laser with a maximum power of 145 mW. UV–VIS transmittance spectra were recorded with a UV-2401PC (Shimadzu Corporation, Kyoto, Japan) spectrophotometer. Electrochemical experiments were performed using a PGSTAT302N Autolab (Metrohm AG, Herisau, Switzerland) potentiostat/galvanostat in a three-electrode cell setup using as the working electrode the V_2_O_5_ layer onto the Fluorine-doped tin oxide (FTO) coated glass substrate, the Pt counter electrode and the reference electrode (Ag/AgCl), and 1 M, LiClO_4_ in propylene carbonate as electrolyte.

## 3. Results and Discussions

### 3.1. X-ray Diffraction Analysis

**Figure 1:**
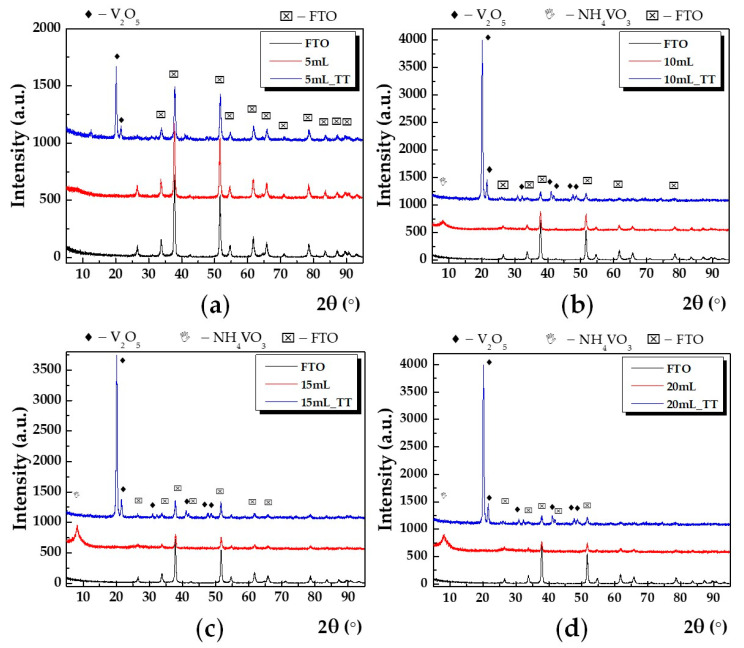
Grazing incidence X-ray diffraction (GIXRD) patterns for: (**a**) 5 mL before and after annealing; (**b**) 10 mL before and after annealing; (**c**) 15 mL before and after annealing; (**d**) 20 mL before and after annealing; with red and blue line, respectively. In addition, GIXRD pattern corresponding to FTO (Fluorine-doped tin oxide) was added (black line).

Grazing incidence X-ray diffraction at small incidence angle (e.g., 0.5°) was used to reveal the crystalline structure of thin films. In Figure 1 are shown GIXRD patterns for the investigated samples: 5, (a), 10, (b), 15, (c) and 20 mL (d) before and after of the thermal treatment (TT). In addition, the GIXRD pattern for FTO substrate (black line) was added.

The peak indexing was made using ICDD database-International Center for Diffraction Database with different symbols, and, namely, V_2_O_5_ with diamond, FTO with star, and NH_4_VO_3_ with triangle. One can observe that the specific diffraction peaks for V_2_O_5_ are absent in the case of all as untreated samples. Thus, all the diffraction peaks are given by the FTO crystalline substrate, except the diffraction peak located at small angle (e.g., around 2θ = 10°), which was attributed to the presence of nonreacted NH_4_VO_3_ and amorphous vanadium oxide. After annealing, the presence of the α-V_2_O_5_ phase was identified according to card no. 01-0359. The Bragg’s law was applied to get the lattice parameters of the unit cell a, b, and c. The obtained values, as well as the unit cell volume are listed in Table 1.

It can be observed that the obtained values for the lattice parameters present small deviations besides to α-V_2_O_5_ with orthorhombic lattice symmetry having parameters a = 11.7 Å, b = 4.40 Å, and c = 3.45 Å, while α = β = γ = 90°, which belongs to the 47:Pmmm space group. This involves the presence of the small lattice strain in each case, which can be observed that led to an expansion of the lattice according to Table 1. Moreover, the diffraction peak corresponding to NH_4_VO_3_ disappeared after the thermal treatment, proving that the full conversion from NH_4_VO_3_ to V_2_O_5_ took place. Further, to study the crystal quality of the obtained V_2_O_5_, we have analyzed the full width at half maximum (FWHM) of the diffraction peaks of our samples. One of the common ways to attest the crystalline quality is Scherrer formula. However, it considers only the effect of crystallite size on the diffraction peak broadening and does not tell anything about the intrinsic strain of the lattice [15] In this sense, we employed Williamson-Hall method [16] that is able to offer a separate description of the crystallite size and the lattice strain. This assumes that the diffraction peak has two components, namely, size broadening, *β_L_*, and strain broadening, *β_S_*, and the dependence of them with respect to Bragg angle, *θ* is:

(1) $$\beta_{\tau }=\;\frac{k\lambda }{\tau cos\theta }\;\beta_{S}=4\varepsilon tan\theta ,$$

where *k* is a shape factor of the crystallites taken as 0.9, *λ* is the X-ray wavelength (e.g., 1.54 Å), *τ* is the mean crystallite size, and ε is the lattice strain.

From Equation (1), it can be observed that the size broadening depends on 1cosθ, while the strain broadening depends on tan*θ*. Essentially, Williamson-Hall method assumes that size and strain contributions are additive factors of the total breadth of the diffraction peak [17,18], thus the convolution is either a simple sum or sum of squares. Combining Equation (1), we obtained:

(2) $$\beta =\;\beta_{\tau }+\beta_{S}=\frac{k\lambda }{\tau cos\theta }+4\varepsilon tan\theta$$

and multiplying this equation by *cosθ* we get:

(3) $$\beta cos\theta =\frac{k\lambda }{\tau }+4\varepsilon sin\theta .$$

**Figure 2:**
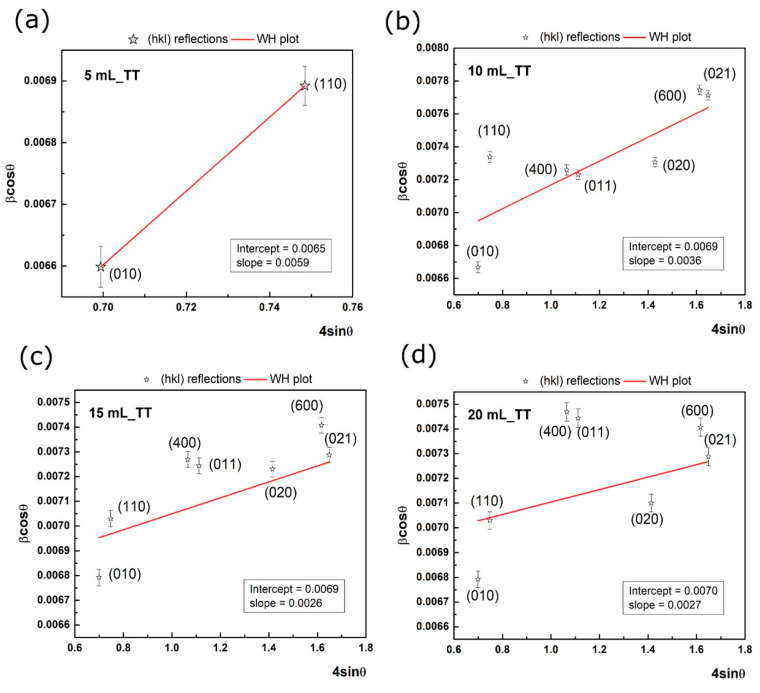
Williamson-Hall plot for: (**a**) 5 mL after annealing, (**b**) 10 mL after annealing, (**c**) 15 mL after annealing, and (**d**) 20 mL after annealing with red line. The values of the intercept and slope are listed in the inset.

It is clear that the intercept may be used to determine the mean crystallite size, while the slope determines the strain term. Since the samples exhibit a high dispersion of the data, it is mandatory to use all reflections given by V_2_O_5_. In particular, high angle reflections are important, since these are sensitive to lattice strain [19], Figure 2a–d presents the Williamson-Hall plots (red line) on multiple reflections for each sample with corresponding intercept and slope values as shown in inset.

To evaluate the errors given by each reflection, the peak broadening was considered as the convolution of two parts, i.e., physical broadening and instrumental broadening [20,21]. Hence, in our case, the errors can be arisen from the instrumental broadening, which was established from a measurement of a monocrystalline Si wafer. The standard deviation of the experimental points was calculated and added on the graph. It can be observed that the experimental points present low deviations. However, the obtained e intercept value showed that the mean crystallite size has the following values: 21.3 ± 0.3 nm (5 mL), 20.1 ± 0.3 nm (10 and 15 mL), and 19.8 ± 0.3 nm (20 mL). The positive slope of the linear fit indicates an expansion of the lattice. In essence, this fact is showed above (see Table 1), where a greater volume of the unit cell is obtained (up to 2.09% in the case of 5 mL_TT sample) compared to the theoretical unit cell volume for α-V_2_O_5_. In addition, this sample presents the highest value for the lattice strain according to W-H analysis. The results obtained from XRD indicate that the thermal treatment led to V_2_O_5_ crystalline thin films with the crystallite size around of 20 nm, accompanied by a small lattice strain.

### 3.2. Morphological Characterization

**Figure 3:**
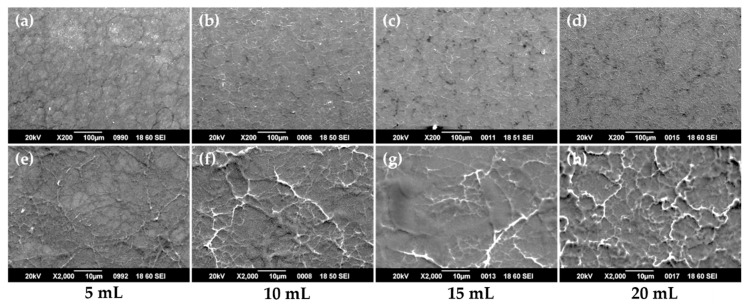
SEM images of the as-deposited films, before annealing (**a**–**d**) SEM micrographs of the 5, 10, 15, and 20 mL precursor quantities deposited films at 200× magnification (scale size 100 ¼m); (**e**–**h**) SEM micrographs of the 5, 10, 15, and 20 mL precursor quantities deposited films at 2000× magnification (scale size 10 ¼m).

SEM characterization of the as-deposited films revealed the films surface evolution as shown in Figure 3. Figure 3a–d presents low magnification SEM micrographs of the 5, 10, 15, and 20 mL precursor quantities deposited films. One can see that films are uniform and homogeneous, free of cracks. A closer view to their surfaces, shown in Figure 3e–h, reveals the presence of random grains-walls structures uniformly distributed onto the surface. Increased precursor quantity (that can be associated to films thickness) seems to determine the increase in wall-like structures density.

**Figure 4:**
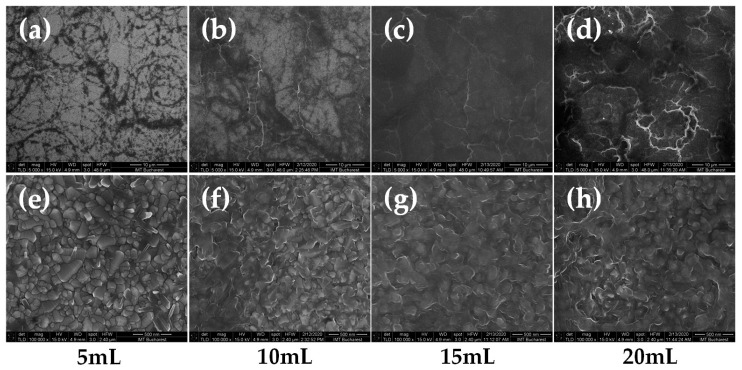
Field-emission scanning electron microscope (FE-SEM) images of the nanostructured V_2_O_5_ films after annealing (**a**–**d**) FE-SEM micrographs of the 5, 10, 15, and 20 mL precursor quantities deposited films at 5000× magnification (scale size 10 ¼m); (**e**–**h**) FE-SEM micrographs of the 5, 10, 15, and 20 mL precursor quantities deposited films at 100,000× magnification (scale size 500 nm).

Annealing have a dramatical effect on films surface evolution. Figure 4 presents HR-SEM images of the same films after the thermal treatment. Figure 4a–d depicts the 5000× magnification of the 5, 10, 15, and 20 mL precursor quantities deposited films presented also in Figure 3, before annealing. It can be observed that, generally, the films preserved the grains and walls-like structuring but the size of grains and walls-like structures quite decreased and analysis to higher magnifications using the FE-SEM was needed. Figure 4e–h shows high-resolution 100,000× magnification images of the granular structuring of the film’s surfaces.

One can see that the thinnest films present a compact granular structuring. As the thickness increases, the granular multilayer structuring appears and evolve leading to a grains-and-voids three-dimensional structure. The film structuring evolves from compact granular packaging in the 5 mL sample case to a structure of granular layers that form the film by overlapping. The grains size forming the layers slightly decreases with increasing precursor volume. As the precursor volume increases, the films seem to have a “fluffier” postannealing structuring. This suggests the increased active surface availability for ionic insertion and makes these films suitable for electrochromic, sensing, and other applications requiring high surface to volume ratio materials [22]. Further AFM studies and surface characteristic parameters would be performed in order to measure the surface to volume ratio evolution. These observations correlate well with the XRD analysis as well as with the Raman spectroscopy studies presented below. It is worth mentioning that, to our knowledge, such kind of V_2_O_5_ layers structuring was not presented in the literature by other research groups.

### 3.3. Raman Analysis

**Figure 5:**
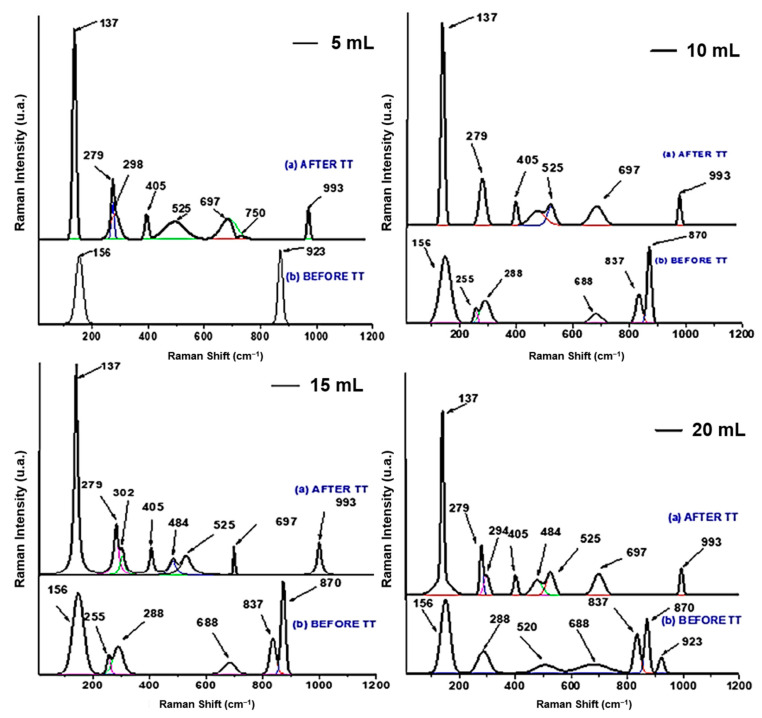
Raman spectra of 0.01 M and 5, 10, 15, and 20 mL samples, before annealing and after annealing.

Figure 5 shows Raman spectroscopic measurements for ammonium metavanadate NH_4_VO_3_ sprayed on the samples at a substrate temperature of 250 °C; measurements were performed in the wavelength range of 10 cm^−1^ to 1200 cm^−1^ and the spectra analysis and peaks identification is synthesized in Table 2. Raman spectra analysis shows that The V_2_O_5_ layers are formed from packing of edge shared VO_x_ square pyramids linked in the “ΧΥ” plane [23]. Orthorhombic V_2_O_5_ system is made of distorted VO_x_ pyramids sharing edges and corners having space group Pmmn (𝐷132ℎ). In the structure, there are three differently coordinated O atoms, OI—vanadyl, OII—bridging, and OIII—chain. The low frequency modes correspond to the X, Y, and Z displacements of the complete chain involving shear and rotations of V-OIII bonds. The highest vibration mode observed at 137–156 cm^−1^ peaks indicates the shear-like distortions of O=O vanadyl bonds (A1g and B2g lines) in-phase rotation around the XY plane. The band at 279 cm^−1^ (B2g/B3g) corresponds to the vibration with a maximum amplitude for OI atoms moving along Y axe, and the displacement of OII atoms along Z axis is assign to Raman peak at 298 cm^−1^. Displacement of OII atoms parallel and perpendicular to “XY” plane generates vibration modes at 697 cm^−1^ (V-OIII-V antiphase stretching mode) and 525 cm^−1^, respectively, which are assigned to V-OIII-V symmetrical and asymmetrical bridges to B1g and B3g.

The V-O-V bridging bond corresponds to the B2g at 923 cm^−1^ and 870 cm^−1^ appears due to the stretching vibrational mode of V-O bond along Z direction. After annealing, the symmetric stretching of the V-O-V bridging bond and angle-bending corresponding to the A1g vibration consolidates its position and are observed experimentally at 405 and 484 cm^−1^. The most affected modes by the temperature in the annealing process are the V=O stretching bands situated at 137 cm^−1^, which has a slight deviation to the left, and the band situated at 525 cm^−1^ increases in Raman intensity. The peak at 850 cm^−1^ are assign to stretching mode of V-OII bonds corresponding to the displacement of OII atoms along X direction. The other V_2_O_5_ vibration modes are affected by the compressive stress introduced by temperature; the modes situated at 870 cm^−1^ disappear and V=O stretching mode at 923 cm^−1^ have an important upshift to 993 cm^−1^ [24].

The Raman analysis correlates with the XRD structural evolution observations.

### 3.4. Optical Spectroscopy and Electrochromic Behavior

**Figure 6:**
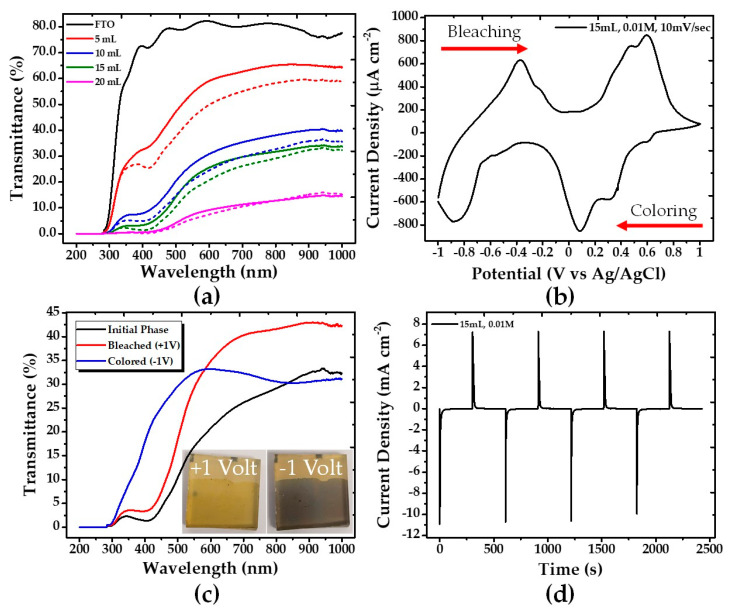
(**a**) UV–VIS transmittance comparison of the samples before (solid lines) and after (dashed lines) annealing; (**b**) Cyclic voltammogram after annealing of 15 mL sample; (**c**) UV–VIS transmittance comparison of the initial, bleached, and colored state of the 15 mL V_2_O_5_ coating; and (**d**) Chronoamperometry after annealing of 15 mL sample.

To investigate the material’s behavior before and after annealing, UV–VIS spectroscopy was employed. The UV–VIS spectra of the same films presented above are shown in Figure 6a. One can observe the expected differentiation in the optical transmittance of the V_2_O_5_ films depending on the volume of precursor’s solution that was used to grow the films in the deposition process correlated to film thickness. Initially, as the volume of the solution increases, the transmittance value decreases, as the coating becomes thicker. In relation to the difference in optical transmittance of the material between before (solid lines) and after (dashed lines) annealing, there is a slight variation of the curves in general, with a slight decrease in the value of the transmittance. What is obvious, however, is that the lower the volume of the solution (the thinner the film), the greater the effect of annealing on the transmittance curve (see 5 and 20 mL curves).

The electrochemical experiments were performed in a three-electrode cell, by cycling the potential between −1 and +1 V at a scan rate of 10 mV s^−1^ and using 1 M LiClO_4_ in propylene carbonate as electrolyte. A typical cyclic voltammogram is shown in Figure 6b. The samples, in general, had similar electrochemical performance, and an example corresponding to one of the annealed samples (15 mL, 0.01 M) is shown in Figure 6b–d. The V_2_O_5_ coating (15 mL, 0.01 M) indicates three cathodic peaks at −0.863, +0.084, and +0.325 V and three anodic peaks at −0.375, +0.469, and +0.592 V, which are attributed to Li^+^ intercalation and deintercalation accompanying gain and loss of an e^−^.

To characterize the electrochromicity of the thin films, UV–VIS spectroscopy analyses in the color change phases of the material were performed in combination with the cyclic voltammetry experiments. In the as-deposited samples (before annealing) a poor electrochromic behavior was observed and with a small difference between the colored and the bleached states. In particular, the color varied between light gray/yellow (−1 V) and light yellow (+1 V). After annealing, the color states changed and improved dramatically, being brown (as the one presented in Figure 6c), blue, and a darker gray, as compared to the original colors before the annealing process. Comparing the bleached states, the annealed samples have stronger yellow tint than the samples before the annealing. The color change of the 15 mL, 0.01 M annealed sample is presented in Figure 6c.

To evaluate the time evolution of the coloring/bleaching processes chronoamperometry measurements were carried out. An example is shown in Figure 6d. When a negative voltage (−1.0 V) was applied, the color of the V_2_O_5_ coating changed from yellow to brown. With the opposite applied voltage (+1.0 V), the film returned to its initial state, becoming yellow again. From the I–t measurements, the time response for coloration of the 15 mL sample was found to be 9.71 s and for bleaching being 14.57 s. As can be observed, the sample has a much faster response to coloring than to bleaching. The charge density was calculated by integrating the current density and was found to be −57.37 and 58.33 mC cm^−2^ for coloration and bleaching, respectively. According to these values, the conclusion is that the ions that enter are about the same as those that leave the surface of the sample, so there is no ion retention in the sample.

One important characteristic in electrochromic properties is the coloration efficiency (*CE*) factor, is defined as the change in optical density (Δ*OD*) per unit of charge density (*Q*), and was calculated according to the following Equation (4):

(4) $$CE(\lambda )=\frac{\Delta OD(\lambda )}{Q},$$

where Δ*OD* is the optical density, showing the change in the transmission between the bleached and colored states of the film, that is, Equation (5):

(5) $$\Delta OD(\lambda )=\ln\left(\frac{T_{b}}{T_{c}}\right),$$

where *T_b_* and *T_c_* are the transmittances at bleached and colored state, respectively. *Q* is the charge density and *λ* denotes certain wavelengths, which, for our calculations, were taken at 700 and 900 nm. The *CE* for our film was calculated to be 3.85 cm^−2^ C^−1^ at 700 nm, increasing up to 5.97 cm^−2^ C^−1^ at 900 nm.

## 4. Conclusions

The annealing effect on the properties of electrochromic V_2_O_5_ thin films grown by spray deposition technique at low temperature was studied. It was found out that the as-deposited films are a mixture of nonreacted precursor material and amorphous V_2_O_5_ phases building up in with unique surface morphology consisting of granular and wall-like randomly distributed structures. The surface formation is strongly influenced by films thickness as correlated with the quantity of sprayed precursor solution. Thermal treatment of these films at 400 °C in O_2_ atmosphere for 2 h has a dramatic effect on films structuring and other physical and chemical properties as well as electrochromic behavior. The films consist on walls-like structures and smaller granular covered valleys that can be improved by postdeposition annealing in O_2_ atmosphere. This kind of growth leads to the formation of a large active surface that favors ionic insertion and enhanced electrochromism. It is worth mentioning that, to our knowledge, such kind of V_2_O_5_ layers structuring as well as the low-temperature growth to define the films’ 3D architecture followed by controlled annealing to achieve stoichiometry and consequently, electrochromic properties were not presented in the literature by other research groups.

## Figures and Tables

**Table 1:** The lattice parameters of the unit cell a, b, and c and its volume derived from the Bragg’s relation for each sample. In addition, the lattice parameters of the α-V_2_O_5_ were added.

Sample	a (Å)	b (Å)	c (Å)	V (Å^3^)
5 mL_TT	11.28	4.38	3.67	181.32
10 mL_TT	11.56	4.40	3.50	178.02
15 mL_TT	11.48	4.42	3.52	178.61
20 mL_TT	11.55	4.38	3.56	180.09
α-V_2_O_5_	11.70	4.40	3.45	177.60

**Table 2:** The Raman spectra peaks and their assignments.

Wave Number (cm^−1^)	Assignment	Vibrations Modes
137, 156	Lattice vibration	A1g, B2g
279	Bending vibration of the V=O bonds	B2g, B3g
298	Bending vibration of the V3-O bonds	Ag
405, 484	Bending vibration V-O-V	Ag
525	Stretching vibration of V3-O bonds	Ag
697	Antisymmetric stretching vibration of V-O_(2)_-V	B1g, B3g
837, 880	Stretching vibration of V=O	B1g
923, 993	Stretching vibration of V=O vanadyl bonds	Ag, B1g
